# Fructose-1,6-bisphosphate prevents pulmonary fibrosis by regulating extracellular matrix deposition and inducing phenotype reversal of lung myofibroblasts

**DOI:** 10.1371/journal.pone.0222202

**Published:** 2019-09-11

**Authors:** Henrique Bregolin Dias, Jarbas Rodrigues de Oliveira, Márcio Vinícius Fagundes Donadio, Shioko Kimura

**Affiliations:** 1 Laboratory of Cellular Biophysics and Inflammation, PUCRS, Porto Alegre, RS, Brazil; 2 Laboratory of Metabolism, Center for Cancer Research, National Cancer Institute, National Institutes of Health, Bethesda, MD, United States of America; University of Colorado School of Medicine, UNITED STATES

## Abstract

Pulmonary fibrosis (PF) is the result of chronic injury where fibroblasts become activated and secrete large amounts of extracellular matrix (ECM), leading to impaired fibroblasts degradation followed by stiffness and loss of lung function. Fructose-1,6-bisphosphate (FBP), an intermediate of glycolytic pathway, decreases PF development, but the underlying mechanism is unknown. To address this issue, PF was induced *in vivo* using a mouse model, and pulmonary fibroblasts were isolated from healthy and fibrotic animals. In PF model mice, lung function was improved by FBP as revealed by reduced collagen deposition and downregulation of ECM gene expression such as collagens and fibronectin. Fibrotic lung fibroblasts (FLF) treated with FBP for 3 days *in vitro* showed decreased proliferation, contraction, and migration, which are characteristic of myofibroblast to fibroblast phenotype reversal. ECM-related genes and proteins such as collagens, fibronectin and α-smooth muscle actin, were also downregulated in FBP-treated FLF. Moreover, matrix metalloproteinase (MMP) 1, responsible for ECM degradation, was produced only in fibroblasts obtained from healthy lungs (HLF) and FBP did not alter its expression. On the other hand, tissue inhibitor of metalloproteinase (TIMP)-1, a MMP1 inhibitor, and MMP2, related to fibroblast tissue-invasion, were predominantly produced by FLF and FBP was able to downregulate its expression. These results demonstrate that FBP may prevent bleomycin-induced PF development through reduced expression of collagen and other ECM components mediated by a reduced TIMP-1 and MMP2 expression.

## Introduction

Pulmonary fibrosis (PF) is a chronic and progressive lung disease with scarring of lung interstitium and progressive decline of lung function. With an overall survival rate of 20% after 5 years of diagnosis, there is currently no treatment to reverse the fibrotic state and lung transplant remains the best option [1, 2]. In the fibrotic tissue, a remarkable characteristic is the fibrotic foci, where active fibroblast to myofibroblast differentiation occurs. This leads to the production and deposition of the extracellular matrix (ECM) components collagen and fibronectin, and α-smooth muscle actin (α-SMA), an actin isoform responsible for cell-generated mechanical tension [3, 4]. In addition, ECM degradation by metalloproteinases (MMP) is compromised by increased production of its inhibitors, tissue inhibitor of metalloproteinase (TIMP) [5]. Thus, fibroblasts and myofibroblasts are regarded as the main source of ECM present in the fibrotic process [6]. Fibroblast activation can occur in response to growth factors after tissue damage, with increasing cell migration to the wound focus, contractility, and acquiring a high proliferative phenotype [7].

Fructose-1,6-bisphophate (FBP), an intermediate of glycolytic pathway, has shown protective effects in a wide range of pathological situations, including sepsis [8] and acute lung injury [9]. FBP has demonstrated anti-oxidant activity both *in vitro* and *in vivo*. However, conflicting results were also reported regarding this effect [10–13]. An earlier study found that activated liver myofibroblasts, or hepatic stellate cells, underwent a phenotype reversal associated with quiescence, decreased proliferation, and accumulation of lipid droplets when treated *in vitro* with FBP, all of which are characteristics of normal healthy fibroblast [14]. This protective effect of FBP was maintained even when these cells were concomitantly exposed to high doses of free iron, that sustains activation of stellate cells [15]. A previous study on lung fibrosis showed that FBP can prevent bleomycin-induced fibrosis development in mice [16], however, the molecular basis of this effect remains unknown. The aim of this study was to determine how FBP prevents and/or regulates the fibrotic process using an animal model of PF induced by bleomycin *in vivo* and primary fibroblasts isolated from healthy and fibrotic lungs of mice *in vitro*.

## Materials and methods

### Pulmonary fibrosis animal model

Male mice (eight week old, C57Bl/6N, 25-30g) maintained in a 12h light-dark cycle with water and chow *ad libitum*, were divided into four groups; 1) Bleomycin (BLM) group: received intratracheally (IT) 1.2U/kg BLM (Sigma-Aldrich) on day 0 [17], 2) control group: received IT an equal volume of saline on day 0, 3) BLM + FBP group; BLM IT on day 0 and thereafter daily intraperitoneal injection (ip) of 500 mg/kg FBP [16, 18] for 14 days, and 4) FBP group: saline IT on day 0 and thereafter daily ip of FBP. Weight and mortality were monitored daily for 14 days. Intratracheal intubation was carried out using the BioLITE system (BioTex, Inc., Houston, TX). Mice were anesthetized with ketamine (100 mg/kg) and xylazine (10 mg/kg) by ip injection, and a catheter, with the aid of the light of an optical fiber, was inserted into the trachea located between the arytenoid cartilages. The optical fiber light guide was removed with maintaining the catheter in a steady position in the trachea and 50 μl of BLM was added to the catheter. The quick suction of the solution through the catheter indicated its correct location in the trachea. All animal studies were performed after approval by the National Cancer Institute Animal Care and Use Committee.

### Lung function

On day 14, the mice were anesthetized with ketamine (100 mg/kg) and xylazine (20 mg/kg), tracheotomized, cannulated and subjected to lung function analysis with the Legacy FlexiVent System (SCIREQ, Montreal, Canada). To prevent spontaneous breathing during data acquisition and to avoid any interference in the analysis, the mice received the paralytic rocuronium bromide (0.5 mg/kg IP) and were kept ventilated for 5 min at 150 breaths/min before the tests, with a tidal volume of 10 mL/kg and 3 cm H_2_O of positive end-expiratory pressure (PEEP). Through broadband (multifrequency) forced oscillation and, with a constant Phase Model parameter, different compartment of lungs were analyzed and the following measurements obtained: tissue elastance (H), which reflects parenchyma stiffness and is measured by energy conservation in the lungs; tissue damping (G) which indicates tissue resistance, is measured by the energy dissipation in the lungs, and reflects the peripheral airways; and Newtonian resistance (Rn), which is the raw resistance of the conducting central airways. For whole thorax measurement, the single frequency forced oscillation maneuver (Snapshot perturbation) was used to obtain values for: compliance (C), which is the easiness with which lungs can be extended; resistance (R), that reflects dynamic lung resistance; and elastance (E), which is the distensibility of the lungs, chest walls and airways, given by elastic rigidity of the lungs. Each perturbation was repeated three times per mouse with a 30 second interval between each measurement.

### Bronchoalveolar lavage fluid (BALF)

After analysis of lung function, the animals were exsanguinated by cardiac puncture and 1 mL of PBS was slowly injected to the lungs through the catheter and immediately aspirated. Samples were centrifuged, supernatant was stored at -80°C and the pellet was resuspended in 250 μl of PBS. The total number of cells were assessed by direct counting in hemocytometer with the Trypan blue exclusion method. For cell differentiation analysis, 200 μL of cell suspension was added in a cytospin slide chamber (Shandon EZ Double Cytofunnel, Thermo Scientific, USA), spun at 800 rpm for 5 min in a Cytospin 4 (Thermo Scientific, USA) and stained with Stain Set Diff-Quik (Siemens Healthcare Diagnosis Inc., Newark, NJ, USA). Percentages of macrophages, neutrophils and lymphocytes were obtained and adjusted by total cell number.

### Lung histological analysis

After BALF collection, the right lungs were tied, collected, quick frozen in liquid nitrogen and stored in -80°C for quantitative RT-PCR (qRT-PCR). For histological analysis, left lungs were inflated under 20 cm H_2_O and fixed overnight in 10% phosphate buffered formalin, dehydrated and embedded in paraffin. Sections of 4 μm were stained with hematoxylin and eosin (H&E) or Picro Sirius Red (PS) for collagen fibers. Sections were made from different portions of lung, so that the analysis was congruent with the fibrotic stage of the whole lung. To develop a modified Ashcroft Score, 30 fields in each lung of every mouse was visualized and classified according to Hübner and coworkers [19]. For PS, images from lung sections were obtained with BZ-X7000 Analyzer (Keyence, Japan) and the area of the tissue stained with PS was accessed with Image Pro Plus® 6.0 software (Media Cybernetics, Inc., Rockville, MD, USA).

### Lung fibroblasts primary culture

Mouse whole lungs were minced and digested with 1 mg/ml collagenase (Gibco, Invitrogen) in RPMI-1640 medium with 2% antibiotic/antimycotic (Lonza) at 37°C. After 1h, dispersed cells were centrifuged, washed in PBS, seeded in 10-cm^2^ dishes with RPMI-1640 containing 1% antibiotic/antimycotic + 10% fetal bovine serum (FBS), and placed in a cell culture incubator. Media was replaced every day until cells reached confluence. Lung fibroblast primary cultures were obtained from healthy (HLF) and fibrotic (FLF) mice. All experiments were carried out using cells between passages 4 and 8.

### Cell proliferation

Fibroblasts were seeded into 96-well plates (2x10^3^ cell/well) and treated with 0.6 or 1.25 mM FBP dissolved in complete growth media. After 24, 48 and 72 h, media was removed and 10% of Cell Counting Kit-8 (CCK8) (DOJINDO, USA) in RPMI-1640 was added to the wells and allowed to react for 2 h. Absorbance was measured at 450 nm.

### Culture insert migration analysis

Fibroblasts were pre-treated with FBP for 72 h and 10^4^ cells were seeded on cell culture inserts with 8 μm pore size (Falcon, USA) with 1% FBS in RPMI-1640 (low serum media). The insert was placed inside a well of a 24-well plate that contained 20% FBS in RPMI-1640 that was used as chemoattractant to stimulate cells to migrate to the bottom of the insert. FBP was added into the media of the upper chamber at 0 (control), 0.6, or 1.25 mM to examine the effect of FBP on cell migration. After 24 h, the cells inside the insert were mechanically removed and those that migrated to the bottom of the insert were incubated with 10% CCK8 solution in RPMI-1640. The absorbance was obtained, and the migration rate was adjusted by healthy control absorbance.

### Cell contraction in collagen gel assay

Collagen solution (Advanced BioMatrix, USA) was impregnated with 10^5^ cells dissolved in RPMI-1640 media at a final concentration of 0.75 mg/ml collagen, added to a 24-well plate and left to polymerize at 37°C for 1 h. The gels were then detached and soaked in 600 μL of complete growth media containing 0 (control), 0.6, or 1.25 mM FBP and incubated for 24 h. Photographs were taken and the surface area of the gel was measured using ImageJ (http://rsb.info.nih.gov/ij) with the area of an empty well considered the initial area occupied by the gel.

### RNA extraction and quantitative RT-PCR

Total RNA was isolated using TRIzol (Invitrogen), and was converted to cDNA using Superscript II reverse transcriptase (Invitrogen, USA). Quantitative RT-PCR (qRT-PCR) was performed with ABI Prism 7900 Sequence Detection System (Applied Biosystems, Foster City, CA) using SYBR Green FastMix (Quanta Biosciences, Gaithersburg, MD). GAPDH was used as a normalization gene and the standard curve method was used to calculate the relative expression. The PCR conditions used were 50°C for 2 min and 95°C for 10 min followed by 95°C for 15 s and 60°C for 40 s for 40 cycles. Primers used for qRT-PCR analysis are shown in S1 Table.

### Western blotting

Total protein was obtained using cold RIPA lysis buffer (Millipore, USA) with protease inhibitor cocktail (Roche Diagnosis, Indianapolis IN), fractionated by SDS-PAGE and transferred to a PVDF membrane using a transfer apparatus according to the manufacturer’s instructions (Bio-Rad, Hercules, CA). After blocking with 5% nonfat milk in TBS (Tris-buffered saline, pH 7.4) for 1 h, the membrane was washed thrice in TBST (TBS + 0.1% Tween 20) and incubated with the following primary antibodies; anti-α-smooth muscle actin (α-SMA, dilution 1:2000), anti-Collagen type 1 (1:1000), anti-MMP1 (1:500), anti-MMP2 (1:500), anti-TIMP-1 (1:500), anti-TIMP-4 (1:500), and anti-β-actin (1:5000). All antibodies were obtained from Proteintech Group-Fisher Scientific, (Hampton, NH). Membranes were washed three times and incubated with HRP linked anti-Rabbit IgG antibody (1:1000, Cell Signaling, USA) for 1 h. Bands were revealed with SuperSignal West Dura kit (Thermo Scientific, Waltham, MA), visualized with ChemiDoc MP Imaging System (Bio-Rad), and normalized by intensity of β-actin bands.

### Statistical analysis

Data were reported as mean ± SD. Analysis of survival was performed by Kaplan-Meier analysis with log-rank (Mantel-Cox). Comparison between two groups was performed by unpaired two-tailed student’s *t*-test. Multigroup comparisons were performed by One-way or Two-way ANOVA, as indicated in each separate analysis, followed by Tukey’s post hoc test using Prism Software (GraphPad Software, v.7, San Diego, CA). A *p* value <0.05 was considered statistically significant in all analysis. Replicates consisted of at least three independent experiments that were performed at different days. The numbers of biological replicates are indicated in each figure legend.

## Results

### Fructose-1,6-bisphosphate prevents pulmonary fibrosis development and collagen deposition in the lungs

**Fig 1:**
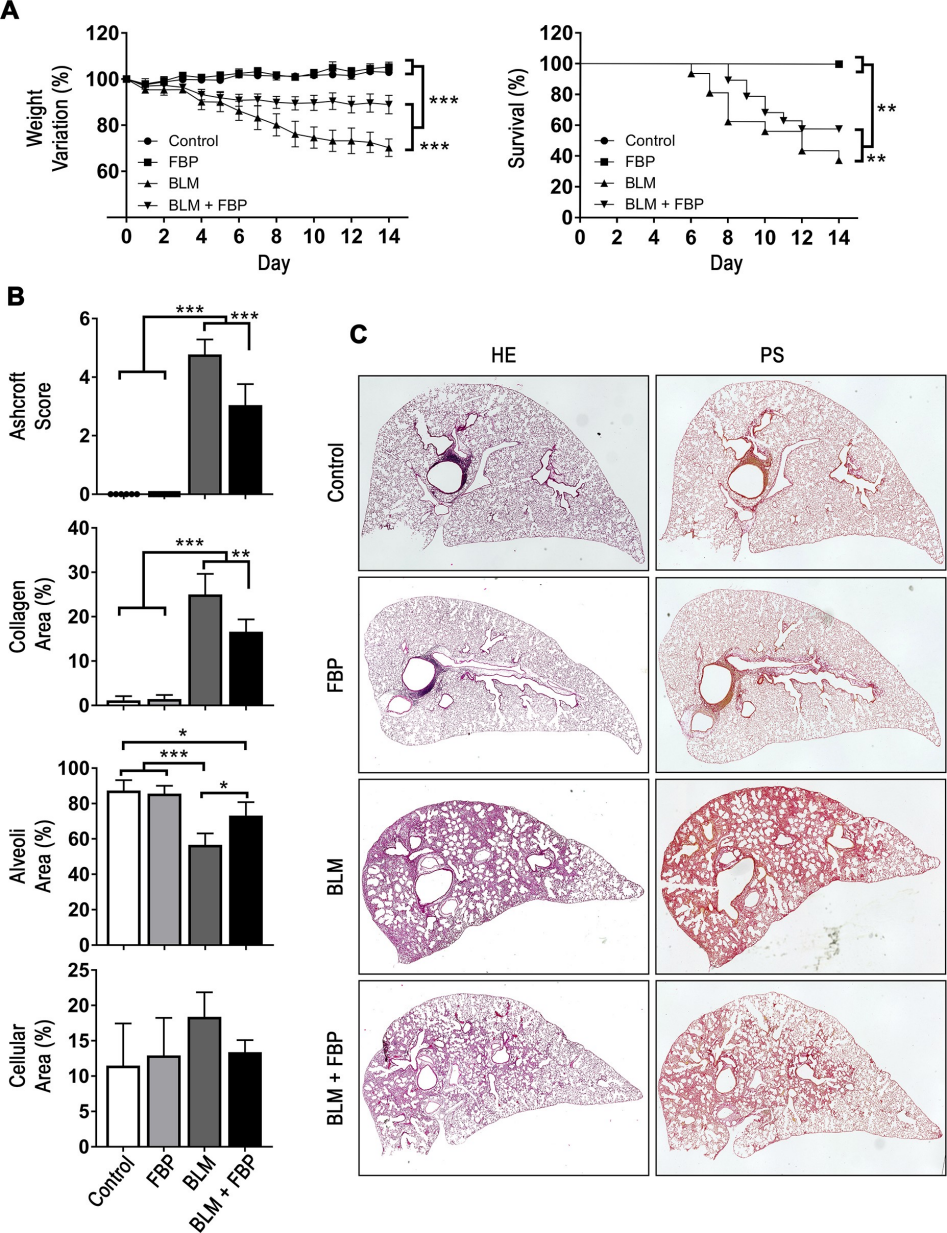
Fructose-1,6-bisphosphate prevents pulmonary fibrosis development and collagen deposition in the lungs. (A) Body weight variation (%) and survival rate (%) are shown during 14 days post-BLM treatment. (B) Ashcroft modified score was used to quantify fibrosis and architecture distortion degree while morphometric analysis was performed to assess area of collagen deposition in the lungs after BLM intratracheal administration and FBP treatment. (C) Representative images of lung sections stained with hematoxylin and eosin (HE) or Picro Sirius Red (PS) showing a high parenchyma distortion and collagen content in the lung after BLM administration. Treatment with FBP shows a lower degree of fibrosis and collagen. Groups’ description given for the whole panel B is on the bottom of the graph showing Cellular Area (%). Weight variation (%) was analyzed by Two-way ANOVA followed by Tukey’s multiple comparison test. Values are expressed as mean ± SD. Survival rate (%) was analyzed by Kaplan-Meier followed by log-rank (Mantel-Cox) test. Ashcroft and morphometric analysis were analyzed by One-way ANOVA followed by Tukey’s multiple comparison test (B). Values are expressed as mean ± SD; For weight variation and survival analysis, n = 6–20/group. For Ashcroft and morphometric analysis, n = 6–12/group; *p<0.05; **p<0.01; ***p<0.001.

Weight loss and mortality are well-known characteristics of the BLM-induced PF model once the fibrotic process starts. BLM induced a statistically significant decrease of body weight in the BLM and BLM+FBP groups when compared to the control and FBP groups (*p*<0.001) (Fig 1A). However, weight loss of the BLM+FBP-treated group was significantly smaller than BLM group (*p*<0.001). All mice from control and FBP groups survived until the end of induction period (14 days). On the other hand, mice from BLM group presented a survival rate of only 38%, which is significantly lower than the 58% found in BLM+FBP group.

To evaluate the extent of PF damage and collagen deposition induced by BLM and the effects of FBP treatment, Ashcroft score and morphometric analysis were performed, which evaluates parenchyma changes and collagen deposition, respectively (Fig 1B). The group that was treated with FBP once a day (BLM+FBP), when compared with non-treated (BLM) mice, had a decreased Ashcroft score, while no changes on lung architecture was observed in the control and FBP-only groups. BLM group also presented a significantly increased percentage of collagen area in the lungs, while a decreased percentage of alveoli space. In comparison, FBP treatment was able to prevent these effects, decreasing the amount of deposited collagen on the parenchyma and preserving the alveolar structure. Although BLM group showed a tendency for increases in cellular area, this value was not different from other groups. The representative histological images of lung sections stained with HE and PS showed a decreased distortion in lung architecture in the BLM+FBP group when compared with mice that received BLM only (Fig 1C). Taken together, these results show that daily treatment with FBP (500 mg/kg) can prevent body weight loss, increase survival and restore the lung architecture of mice with PF induced by BLM.

### Fructose-1,6-bisphosphate decreases inflammatory cell migration and improves lung function

**Fig 2:**
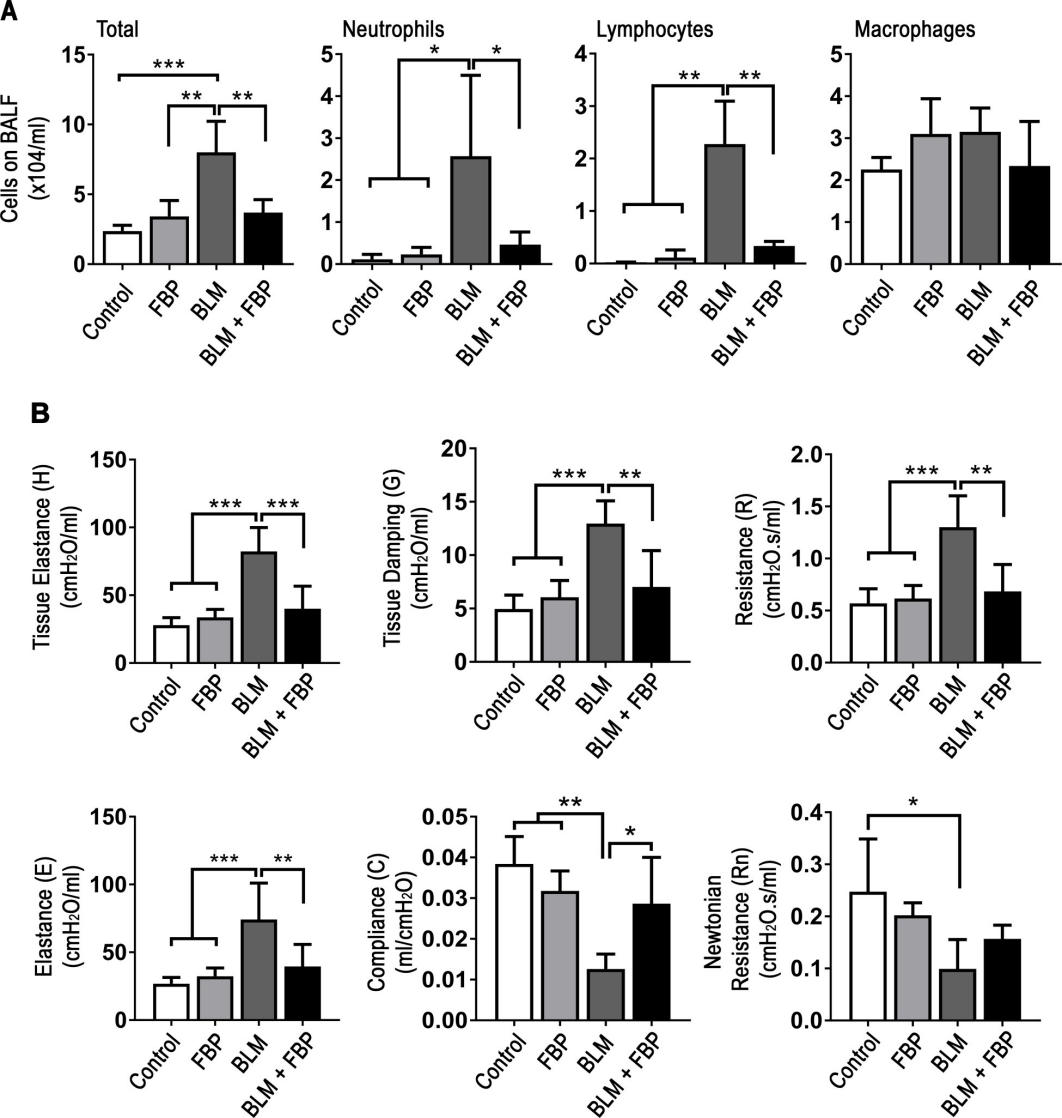
Fructose-1,6-bisphosphate decreases inflammatory cell migration to the lungs and improves lung function. (A) Total number of cells, neutrophils, lymphocytes, and macrophages in BALF assessed by direct counting in hemocytometer. (B) Lung function measurement for tissue elastance (H, parenchyma stiffness), tissue damping (G, tissue resistance that reflects the peripheral airways), resistance (R, level of constriction in the lungs), elastance (E, stiffness of respiratory system), compliance (C, easiness with which respiratory system can be extended), and Newtonian resistance (Rn, reflects the resistance of central and conducting airways). One-way ANOVA followed by Tukey’s multiple comparison test was used. Values are expressed as mean ± SD; n = 6–12/group; *p<0.05; **p<0.01; ***p<0.001.

BLM induces a strong inflammatory response in lungs and, consequently, many leucocytes are recruited to the tissue. BALF was collected to evaluate whether FBP influences immune cell migration. The total number of cells in the lung was significantly increased in animals that received BLM when compared to control, FBP, and BLM+FBP groups (Fig 2A). This effect was due to an increased number of neutrophils and lymphocytes, however the number of macrophages did not differ between the groups.

Fibrosis also increase tissue stiffness, which leads to lung function loss. Accordingly, intratracheal BLM administration caused a decrease in overall lung function, as illustrated by increased on tissue elastance (H), tissue damping (G), resistance (R) and elastance (E), while lowered compliance (C). For Newtonian resistance (Rn) parameter, BLM group was lower only when compared to Control group (Fig 2B). BLM+FBP group had lung function range similar to Control and FBP groups in all tested criterions.

### Fructose-1,6-bisphosphate regulates extracellular matrix-related genes *in vivo*

**Fig 3:**
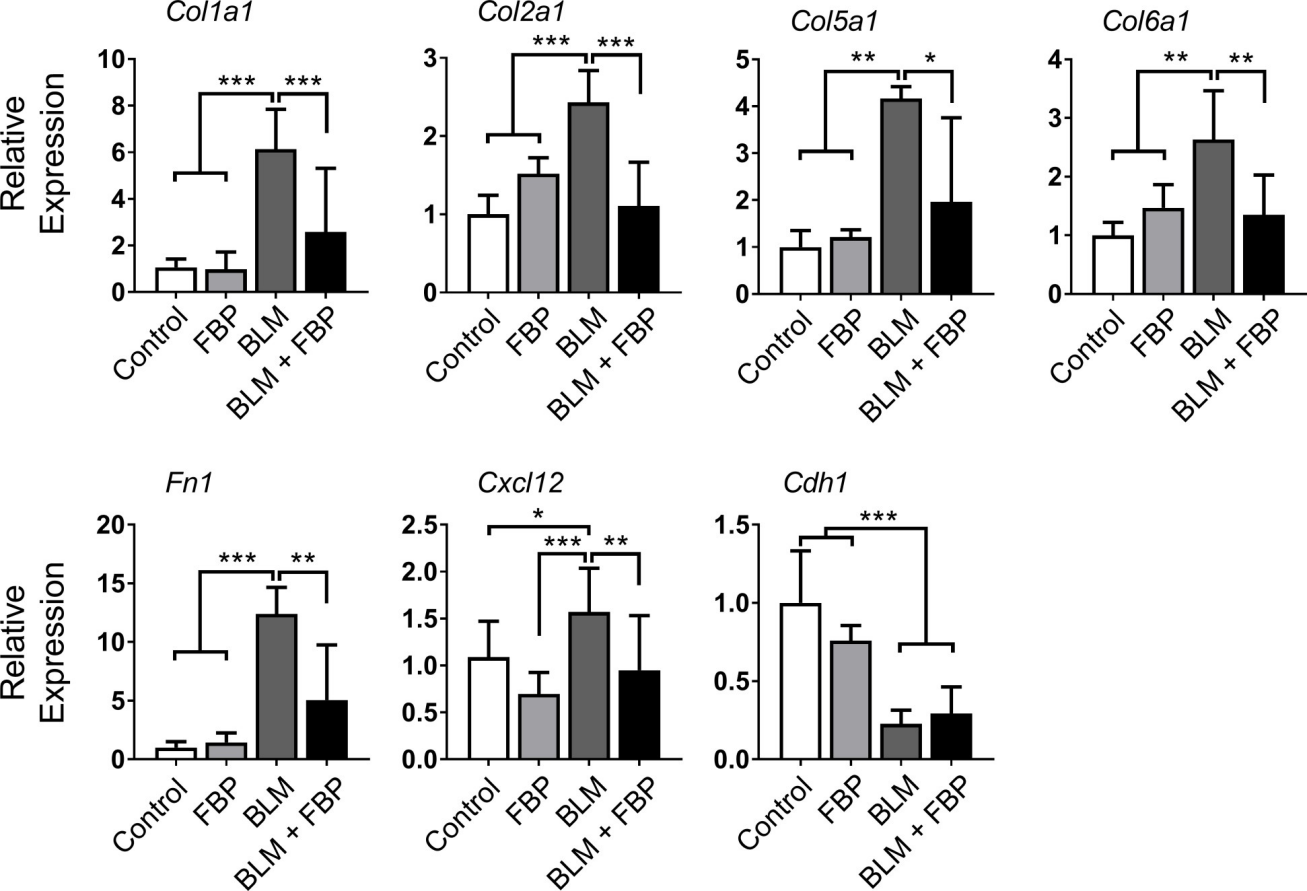
Fructose-1,6-bisphosphate regulates extracellular matrix-related genes *in vivo*. qRT-PCR analysis of mRNA encoding for collagen subtypes, collagen 1a1 (*Col1a1*), collagen 2a1 (*Col2a1*), collagen 5a1 (*Col5a1*), collagen 6a1 (*Col6a1*), and *Fn1*, *Cxcl12* and *Cdh1*. One-way ANOVA followed by Tukey’s multiple comparison test was used. Relative expression is presented based on the expression level of control as 1. Values are expressed as mean ± SD; *n* = 6–12/group; *p<0.05; **p<0.01; ***p<0.001.

The fibrotic process leads to the upregulation of many ECM genes. Thus, *Col1a1*, *Col2a1*, *Col5a1* and *Col6a1* mRNAs were measured. All mRNAs were increased 14 days after BLM intratracheal administration when compared to the control, FBP, and BLM + FBP groups (Fig 3). The mRNAs encoding fibronectin (*Fn1*), another important ECM component, and C-X-C motif chemokine ligand 12 (*Cxcl12*), a fibrosis-associated chemokine, were also increased by BLM administration and FBP was able to downregulate the expression of these mRNAs. Further, BLM decreased *Cdh1* (E-cadherin) mRNA levels, which is inversely associated with the EMT process, and was not affected by treatment with FBP.

### Fructose-1,6-bisphosphate induces deactivation of fibroblasts *in vitro*

**Fig 4:**
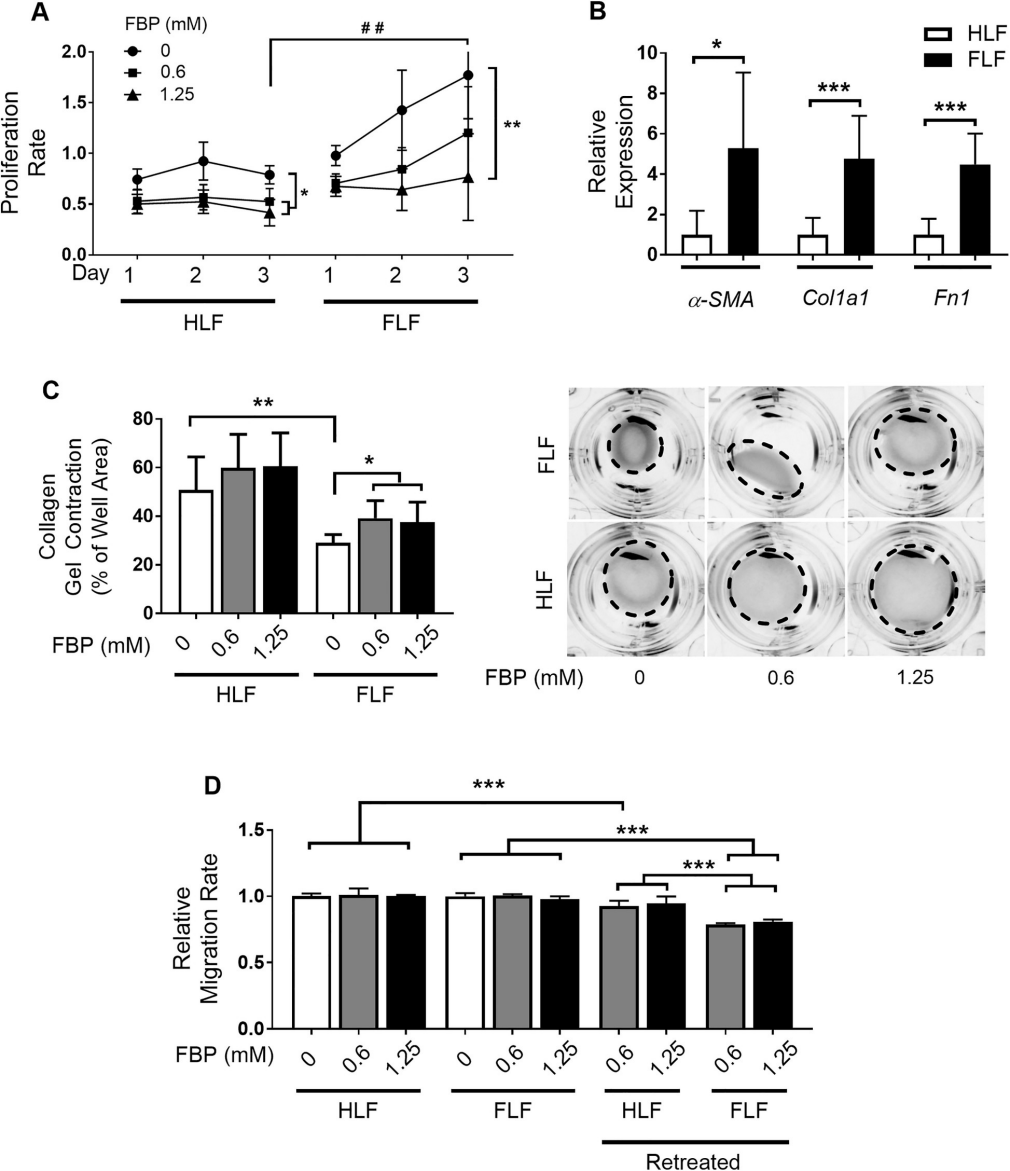
Fructose-1,6-bisphosphate induces non-activated phenotype in fibroblasts. (A) Cell growth. Cells were treated with 0, 0.6, and 1.25 mM FBP and proliferation rate of fibroblasts from healthy (HLF) and fibrotic (FLF) lungs was assessed in 1, 2, and 3 days after treatment. Y axis shows the absorbance of CCK8 assay. (B) Comparison of ECM components gene expression between cells extracted from healthy (HLF) and fibrotic (FLF) lung tissues. (C) Cell contraction analysis in collagen gel. Cells were embedded in a collagen solution and allowed to polymerize to form a gel. The gel was detached from the well and treated with different concentrations of FBP. Results are expressed as a percentage of well area occupied by the gel after a 24 h of incubation based on the size of empty well considered as 100%. Representative images used for the analysis are shown on the right. Dashed line shows the outline of the contracted gels after 24 h. (D) Cell migration assay. Cells were treated with FBP (0.6 or 1.25mM) for 3 days, detached and seeded inside an insert with 1% FBS RPMI-1640. The insert was placed inside a well of a 24-well plate with 20% FBS as chemoattractant and the invasion migration was assessed 24 h later. For retreatment, fresh FBP solution was added to the cells inside the insert (1% FBS RPMI-1640 + 0.6 or 1.25 mM FBP). The relative migration rate for all groups was calculated considering the migration values of non-treated healthy fibroblasts (HLF 0 mM FBP) as 1. Two-way ANOVA followed by Tukey’s multiple comparison test was used in A, unpaired two-tailed student’s t-test was used in B, and One-way ANOVA followed by Tukey’s multiple comparison test was used in C and D. Values are expressed as mean ± SD; n = 4–6/group; ## p<0.01 Healthy (HLF) vs. Fibrotic (FLF) without FBP (Control group) on Day 3; *p<0.05; **p<0.01; ***p<0.001.

When activated, myofibroblasts acquire a high proliferative phenotype. In order to determine whether FBP interferes with the proliferation rate of primary fibroblasts, cells extracted from healthy (HLF) and fibrotic (FLF) mouse lungs were subjected to FBP treatment. FLF had a significantly higher proliferation rate than HLF and treatment with FBP slowed the proliferation rate of both FLF and HLF after three days of treatment with 0.6 and 1.25 mM FBP (Fig 4A). These two doses were chosen because the lower dose of 0.3 mM FBP did not show significantly different effects after three days, while the higher doses of 2.5 to 10 mM FBP were toxic to the cells (S1 Fig). To confirm the nature of HLF and FLF cells, their gene expression profiles were analyzed. The mRNAs encoding all three major ECM components, *α-SMA*, *Col1a1*, and *Fn1*, were higher in cells extracted from mice that were fibrotic (FLF) when compared to those from healthy non-fibrotic mice (HLF) (Fig 4B).

High contractility and EMT process are also important characteristics of myofibroblasts for wound closure and migration to the wound site. In the analysis of contraction on collagen gels, HLF treated with FBP were not different from control cells at any dose tested. However, FLF were more contractile than HLF and FBP-treatment decreased cell contractility at both doses tested (Fig 4C). For insert migration assay, cells were treated with FBP for 3 days. The migration rates of healthy and fibrotic cells were not altered after 24 hours. However, when FBP was maintained for another 24 hours (re-treatment), the migration rates of healthy and fibrotic cells decreased. This effect was more profound in fibrotic cells, and their levels were much lower than healthy re-treated cells (Fig 4D). Together, these results indicate that FBP can influence fibroblast behavior and lead to a deactivated, fibroblast-like phenotype.

### Fructose-1,6-bisphosphate down-regulates collagen-related gene expression *in vitro*

**Fig 5:**
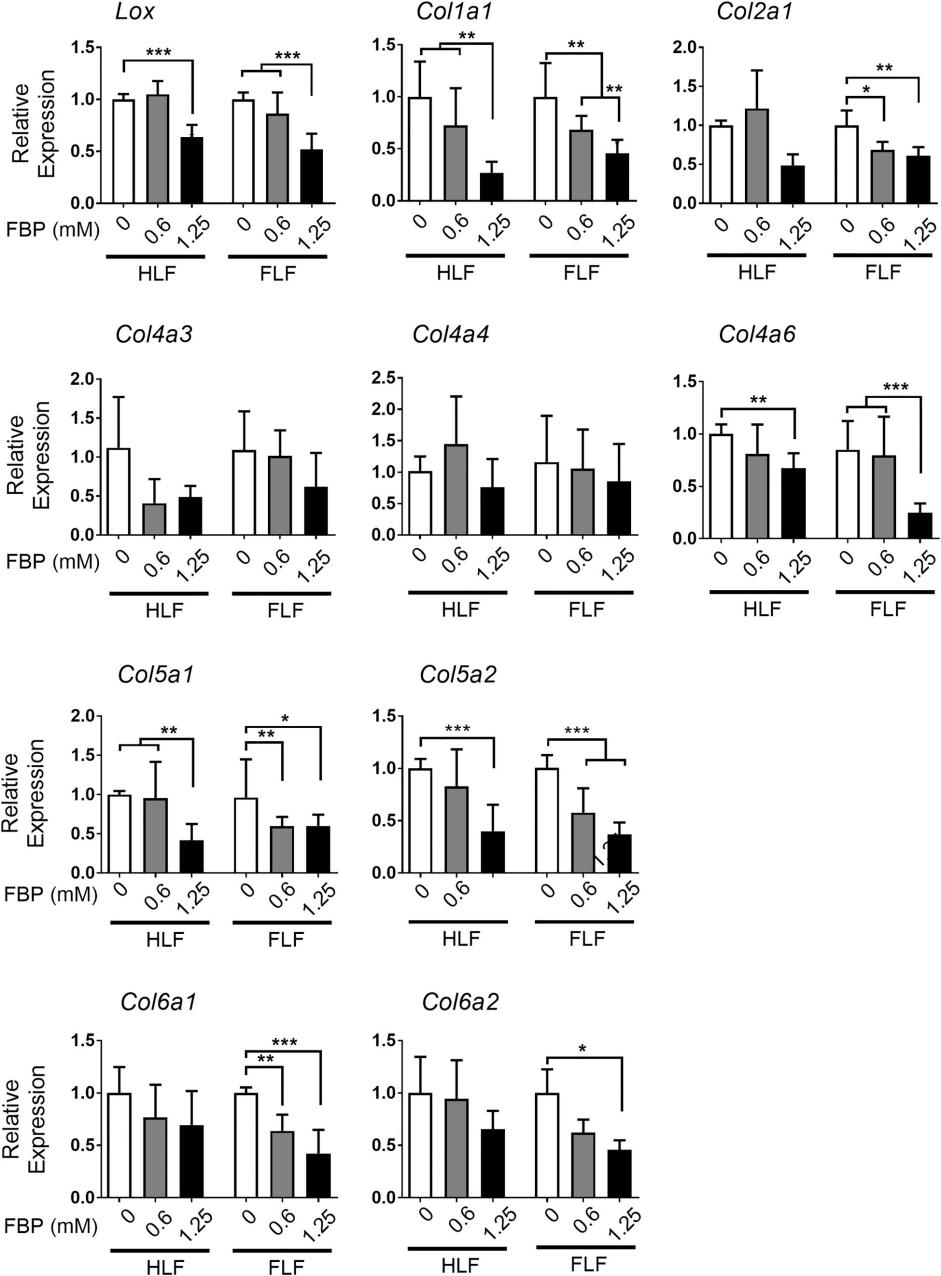
Fructose-1,6-bisphosphate regulates collagen-related gene expression in HLF and FLF *in vitro*. Results show qRT-PCR analysis of mRNAs encoding for lysil oxidase (*Lox*), collagen 1a1 (*Col1a1*), collagen 2a1 (*Col1a2*), collagen 4a3 (*Col4a3*), collagen 4a4 (*Col4a4)*, collagen 4a6 (*Col4a6)*, collagen 5a1 (*Col5a1*), collagen 5a2 (*Col5a2*), collagen 6a1 (*Col6a1*), and collagen 6a2 (*Col6a2)*. Relative expression is presented based on the expression level of control denoted as 1. One-way ANOVA followed by Tukey’s multiple comparison test were used. Values are expressed as mean ± SD; *n* = 4–6/group; *p<0.05; **p<0.01; ***p<0.001.

Since myofibroblasts are source of collagen production and deposition in fibrosis, the expression of collagen subtypes and lysil oxidase (LOX), a protein responsible for cross linking and maturation of collagen fibers [20], was examined using HLF and FLF treated with FBP for 72 h (Fig 5). FBP significantly downregulated expression of *Lox* mRNA and many collagen subtypes, both in HLF and FLF, such as *Col1a1*, *Col4a6*, *Col5a1*, *Col5a2* mRNAs, and this effect was most noticeable with the dose of 1.25 mM FBP. For *Col2a1*, *Col6a1* and *Col6a2* mRNAs, the expression was significantly decreased by FBP treatment only in FLF cells, and not in normal fibroblasts, while the expression of *Col4a3* and *Col4a4* mRNAs was not altered.

### Fructose-1,6-bisphosphate regulates expression of ECM and ECM-degrading proteins *in vitro*

**Fig 6:**
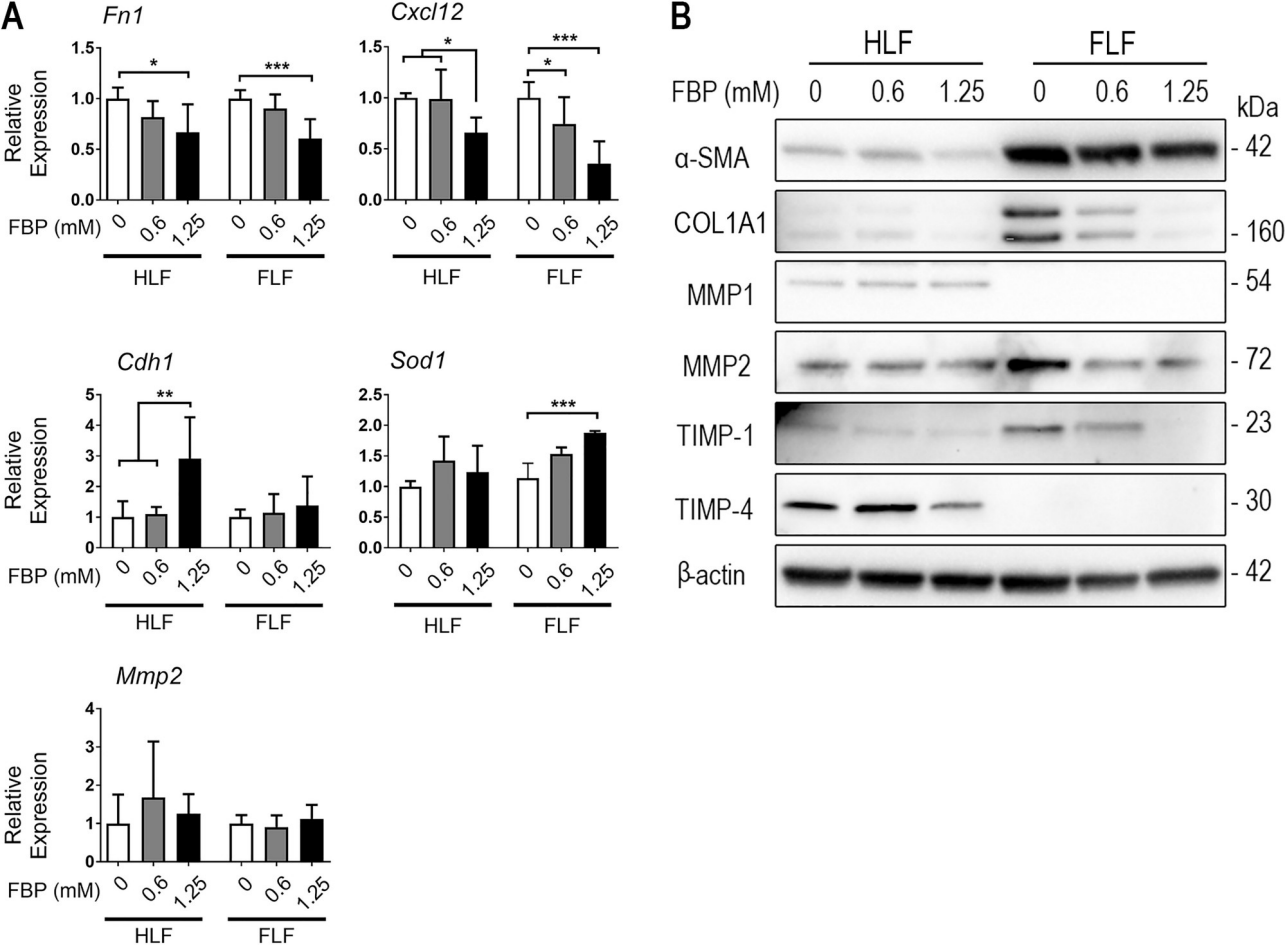
Fructose-1,6-bisphosphate regulates expression of ECM and ECM-degrading proteins *in vitro*. (A) qRT-PCR analysis for the levels of Fn1, Cxcl12, Cdh1, superoxide dismutase 1 (Sod1), and metalloproteinase 2 (Mmp2) mRNAs. Relative expression is presented based on the expression level of control as 1. One-way ANOVA followed by Tukey’s multiple comparison test was used. Values are expressed as mean ± SD; n = 4–6/group; *p<0.05; **p<0.01; ***p<0.001. (B) Protein expression of α-SMA, COL1A1, MMP1, MMP2, TIMP-1, and TIMP-4 in HLF and FLF treated with FBP for 3 days.

Expression of *Fn1* and *Cxcl12* mRNAs were decreased in cells that were treated with FBP while *Cdh1* mRNA expression was increased only in HLF at the higher FBP dose (Fig 6A). FBP was also able to increase superoxide dismutase 1 (*Sod1*) mRNA expression at the higher dose and in FLF cells. *Mmp2* mRNA was not different from control in any cells or FBP doses tested. Myofibroblasts are recognized to be one of the main sources of extracellular matrix in fibrosis and its degradation is compromised through inhibition of MMPs. Immunoblotting showed that, compared to HLF, FLF expressed higher levels of COL1A1, α-SMA, TIMP-1 and MMP2 (Fig 6B). Three days of FBP-treatment clearly decreased the expression levels of COL1A1, MMP2, and TIMP-1 in FLF. MMP1 and TIMP-4 levels were higher in HLF compared to FLF and the treatment with 1.25 mM FBP slightly reduced TIMP-4 expression while FBP had no effect on MMP1.

## Discussion

FBP is a metabolite from glycolysis produced by phosphofructokinase-1, that has many protective properties [13, 16]. In this study, the effects of FBP were evaluated in an experimental model of BLM-induced PF and in *in vitro* studies using lung fibroblasts derived from healthy and fibrotic mice. The results showed that FBP prevents the progression of PF, as revealed by improved lung function in mice, and altered ECM production, deposition and degradation. The *in vitro* results further demonstrated a phenotypical reversion-like phenomenon in lung fibroblasts by FBP treatment.

A previous study [16] showed that a single dose of FBP given at the time of BLM administration can prevent PF, with improvement of lung function. In the current study, mice were administered FBP once a day for 14 consecutive days. This was based on the hypotheses that this treatment schedule could exhibit a stronger protective effect. However, this did not seem to be the case since the mice still presented with considerable fibrosis, as seen by the Ashcroft score, mortality and weight loss. This suggests that the protective effect may be limited. Another possible reason could be the variable ranges of bleomycin activity (10 mg of bleomycin can range through 15 to 20 units) depending on the source of manufacturer. Alternatively, it is known that the fibrotic response to bleomycin is strain-dependent and C57BL/6 mice are more susceptible than BALB/c mice because of different expression of bleomycin hydrolase, cytokines and proteases [21, 22]. Since the genetic background is very important, even different mouse substrains could lead to different experimental observations even under similar conditions [23].

In the earlier studies, a human cell lineage of lung fetal fibroblasts were used [16]. Fetal and adult fibroblasts behave in different ways. Notably, human lung fetal fibroblasts have higher expression of TGF-β1 receptor interacting protein 1, and are less able to contract collagen gels than adult lung fibroblasts [24]. In heart, fetal fibroblasts have more plasma membrane and higher expression of proteins that promote the development and proliferation of cardiomyocytes, whereas adult fibroblasts are more enriched with ECM components [25]. Fetal lung fibroblasts, when confluence is reached *in vitro*, undergo cell cycle arrest even under the stimulation of growth factors, while adult fibroblasts will continue to proliferate [26]. Thus, adult fibroblasts may better reflect the response under pathological conditions, differentiating the characteristics of normal from fibrotic lungs. In the current study, in order to better demonstrate the effect of FBP, we extracted lung fibroblasts from healthy and fibrotic adult mice and treated these cells *in vitro* with FBP for 3 days.

It was thought that FBP loses its phosphates and crosses the membrane as fructose [27]. Another study demonstrated that cells and tissues treated with labelled [C^13^]FBP produce [C^13^]lactate, which indicates that exogenously given FBP can participate in the glycolytic pathway to reestablish ATP pool inside the cells [28]. One hypothesis is that FBP could briefly interact with calcium that stabilizes bilayer lipid membrane, leading to an increase in membrane fluidity that would allow FBP to cross it through passive diffusion [29]. Further, the permeability of FBP through a dicarboxylate transporter was reported, although only the linear form of FBP, a small fraction of total FBP, can use this system [30]. Despite of many authors have confirmed the effect of FBP in a variety of tissues, such as liver [31, 32], kidney [33], brain [34] and lung [9, 16], the exact mechanism of how FBP can enter the cells remains unknown.

The ranges of FBP doses used in our studies were between 12.5 and 15 mg per animal *in vivo* (animals with 25 and 30 g receiving 500 mg/kg, respectively), and 0.212 mg/ml (0.625 mM) or 0.425 mg/ml (1.25 mM) for *in vitro* treatment. A study that analyzed FBP concentration in blood of rats after a single dose of 500 mg/kg injected via IP demonstrated that the peak was reached 2 h after administration, going from control level of 15 to 35 μg/ml, which then came down to 20 μg/ml after 6 h and remained there for at least 72h [35]. This effect was also observed in the brain; the FBP levels peaked at 2 hours after administration (from 0.4 at control level to 0.6 mg of FBP/g of brain tissue), followed by slow decrease during the follow 48 h (to 0.5 mg FBP/g brain tissue). The level stayed above the base line concentration up to 72h. For rats receiving 180 mg/kg FBP orally every 8 h for 7 consecutive days, FBP levels in plasma peaked at 182 μg/ml after 45 min [36]. To our knowledge, there is no report on the amount of FBP captured by lung after IP administration in mice, although reports can be found that it can prevent ischemic lung injury in rats [37]. Based on these, we believe that the amount of FBP we used in *in vitro* experiments is close to *in vivo* physiological range.

Lung function is a hallmark of PF and an important clinical indicator in humans [1, 38]. It reflects the changes in quality and an increase in the quantity of ECM components accumulated during the fibrogenic process, leading to tissue stiffening [39]. Degradation of ECM by MMPs is highly dysregulated in PF and, paradoxically, some MMPs can be overexpressed and linked to a more severe and advanced pathology stage in the lung. This is well known for MMP2, which can disrupt the basement membrane, leading to increased fibroblast invasion of the alveolar spaces. MMP1, an interstitial collagenase, is not as widely distributed in PF lungs as its inhibitor, TIMP-1. TIMPs, that inhibit MMP lytic activity, are present in great amounts in lung parenchyma of IPF patients and animals with induced PF, which contributes to an environment where collagen and other ECM components are not properly degraded [5, 40]. In mice, invasive pulmonary function, used in this study, can exhibit the same functional parameters that are measured in humans and it is the one that better reflects the diseased state of the lung [41]. The present results showed an improvement in lung function by FBP both *in vivo* and in *in vitro* experiments, which is due to FBP regulating the deposition and degradation of many ECM components. Several studies linked levels of production and deposition of collagen and other ECM components with the severity of fibrosis [42]. Cross-linking of collagen fiber is an essential step for collagen maturation and to acquire its mechanical and physical properties. This process is carried out mainly by the LOX enzyme, which is highly increased in PF [39, 42, 43] but decreased in both HLF and FLF treated with FBP. This leads us to believe that FBP may influence collagen maturation and prevent from its uncontrolled deposition.

The aspect of inflammation on human idiopathic pulmonary fibrosis (IPF) is very controversial since no treatment with immunosuppressant was able to improve the clinical course or increase survival rate of patients in clinical trials. However, some authors believe that inflammation is still crucial for IPF development, because inflammatory cells can be seen in lung biopsies and many cytokine levels are higher in BALF from IPF patients [44]. CXCL12 is a cytokine that stimulates leucocyte migration to inflammation site [45] and fibroblast activation [46]. Our data show that FBP-treatment 1) decreases bleomycin-induced pulmonary fibrosis in mice, 2) inhibits inflammatory cell migration to the lungs of mice, and 3) decreases CXCL12 expression levels in healthy and fibrotic mouse lung fibroblasts *in vitro*. These suggest that an inflammatory activity may be involved in pulmonary fibrosis development and FBP plays a role in this process. Based on the expression of ECM components, MMPs, and TIMPs, however, we believe that there may be other mechanisms behind FBP actions than anti-inflammatory activity. Further studies are required to address these questions.

Recently, Zhao and coworkers showed that skin ECM production, including collagen, is related to an upregulation of glycolysis and downregulation of gluconeogenesis, PPARγ expression and fatty acid oxidation, as observed both *in vitro* in dermal primary human fibroblasts and *in vivo* in patients that presented skin fibrosis [47]. On skin equivalents, FBP increased the amount of collagen fibrils on dermis, with increased phosphorylation of p38 MAPK and decreased desmosomal components [48]. Similarly, another study showed that idiopathic pulmonary fibrosis and fibroblast activation are related to an upregulated glycolysis with higher activity of glycolytic enzymes and lactate production [49]. However, a metabolomic analysis on IPF patients showed the contrary; downregulated glycolysis with lower rate of phosphofructokinase (PFK) and lower level of FBP with increased lactate production, reduced mitochondrial beta-oxidation, and downregulated tricarboxylic acid (TCA) cycle in IPF lungs [50]. To proliferate, cells can deviate metabolism from oxidative phosphorylation to aerobic glycolysis, generating lactate, in a phenomenon that is known as Warburg effect. Although less efficient in producing ATP from glucose, it allows cells to divert glucose to produce macromolecular precursors to sustain growth [51]. Even if increased glycolysis is a key element for fibrosis development, it is difficult to know whether glycolysis is the inducer of fibroblast activation, as postulated in some of the studies [47–50]. In this way, it is plausible that Warburg effect happens to sustain the growth and metabolic changes needed after a growth factor signaling, i.e. TGF-β1, and the upregulation of glycolysis would be a consequence, not the trigger, of fibrotic changes in tissues and cells.

Exogenously administered FBP can also act as an activator of glycolysis, increasing lactate production rate [28] and serum ATP [52], but not preserving intracellular pH as shown in cerebral ischemia [53]. As PFK activity is inhibited by high levels of ATP and low pH [54], FBP administration could even result in a halt on PFK activity, leading to an inhibition of glycolytic pathway and consequent downregulation of fibrosis. Others report that FBP could upregulate the expression of PPARγ in hepatic stellate cells, which could increase fatty acid oxidation and decrease glycolysis [15]. Further studies are necessary to determine the exact role of FBP in cell metabolism, which are currently underway.

There are studies supporting that a fully differentiated myofibroblast phenotype is necessary to increase collagen production [55], and the present results showed that cells extracted from the fibrotic lung indeed presents increased proliferation, contraction, invasion and ECM components gene expression. Three days after FBP exposure, both healthy and fibrotic cells were significantly affected by FBP *in vitro*, showing signs of going to a quiescent state that is characteristic of normal fibroblasts. In agreement with these results, previous studies showed that FBP treatment *in vitro* promotes the deactivation of liver fibroblasts [14] even in the presence of free iron that activates fibroblasts to become myofibroblasts [15]. These results mirror some of the *in vivo* experiments, showing that FBP can have protective effects in a more complex system such as intact tissues. While most mechanisms are shared between different organs and tissues, each one represents its own challenge regarding therapeutic approach. Further, it is likely that the *in vitro* experiments presented herein may not be totally extrapolated to the much more complex *in vivo* system. However, it’s interesting to note how FBP can robustly reproduce its effects in different cell lines (lung vs liver) *in vitro* and animals.

Cellular senescence is an irreversible cell-cycle arrest, where the cell maintains a metabolic and secretory profile, and can be induced by many stimuli, including oxidative stress DNA damage [56]. Others showed that FBP induced oxidative stress in hepatic carcinoma cells, which caused an inhibition in proliferation, and this effect was suppressed when these cells were treated with anti-oxidants, such as N-acetyl-l-cysteine or catalase [12]. In contrast, using the same cell line, HepG2, FBP-treatment led to cell senescence, an increase in catalase activity [10] and a decrease in thiobarbituric acid reactive substances, which is a lipid peroxidation product and an indirect measure of radical oxygen species (ROS) [57]. Regardless of this discrepancy, there is a strong body of evidence suggesting that FBP has anti-oxidant properties [13] and, in the present study, FBP was able to increase expression of an antioxidant enzyme, SOD1, in FLF. A study also showed that FBP treatment induced senescence in human lung fetal fibroblasts, which resulted in a lower proliferation rate [16]. This is in good agreement with the present study showing a lower proliferation rate and many other aspects of phenotypical reversion of lung fibroblasts after FBP treatment. Myofibroblasts are initially activated from fibroblasts by various causes including tissue/cell damage. After prolonged damage, myofibroblasts can become senescent, which results in reduced ECM deposition and increased production of ECM degrading enzymes. This phenomena will limit the accumulation of fibrotic tissue and help fibrosis to resolve [58]. In cancer, senescent myofibroblasts express α-SMA and possess a high contractile phenotype. However, their ability to generate organized collagenous matrix is greatly reduced, suggesting that senescent myofibroblasts are α-SMA positive but non-fibrogenic cells [59].

In conclusion, the present results show that FBP prevents BLM-induced PF development in mice. *In vitro* and *in vivo* results demonstrate that this may be due to reduced expression of collagens and other ECM components, which may partly be the consequence of a reduced TIMP-1 and MMP2 expression. However, understanding the exact mechanism for the anti-fibrotic activity of FBP awaits further studies. To our knowledge, this is the first study that demonstrates a regulatory mechanism of FBP in terms of expression of genes and proteins that are responsible for ECM production and degradation. This may open the possibility of evaluating the activity of FBP on patients undergoing cancer treatment as a preventive tool against the harmfull effects of BLM in the lung.

## Supporting information

S1 Fig Fructose-1,6-bisphosphate inhibits healthy lung fibroblasts (HLF). Cells were treated with 0 to 10 mM of FBP and proliferation rate of fibroblasts from healthy (HLF) lungs was assessed through 1 to 7 days after treatment. Y axis shows the proliferation rate as determined by MTT assay. Two-way ANOVA followed by Tukey’s multiple comparison test was used. Values are expressed as mean ± SD; n = 4–6/group; * p<0.05 determined on Day 3.
(TIF)

S1 Table Primer sequences used for qRT-PCR analysis. (DOCX)
