# Supplementary figures and images for: Fructose-1,6-bisphosphate prevents pulmonary fibrosis by regulating extracellular matrix deposition and inducing phenotype reversal of lung myofibroblasts
Source: PLoS One. 2019 Sep 11;14(9):e0222202. doi: 10.1371/journal.pone.0222202 (PMC6738633; doi:10.1371/journal.pone.0222202)

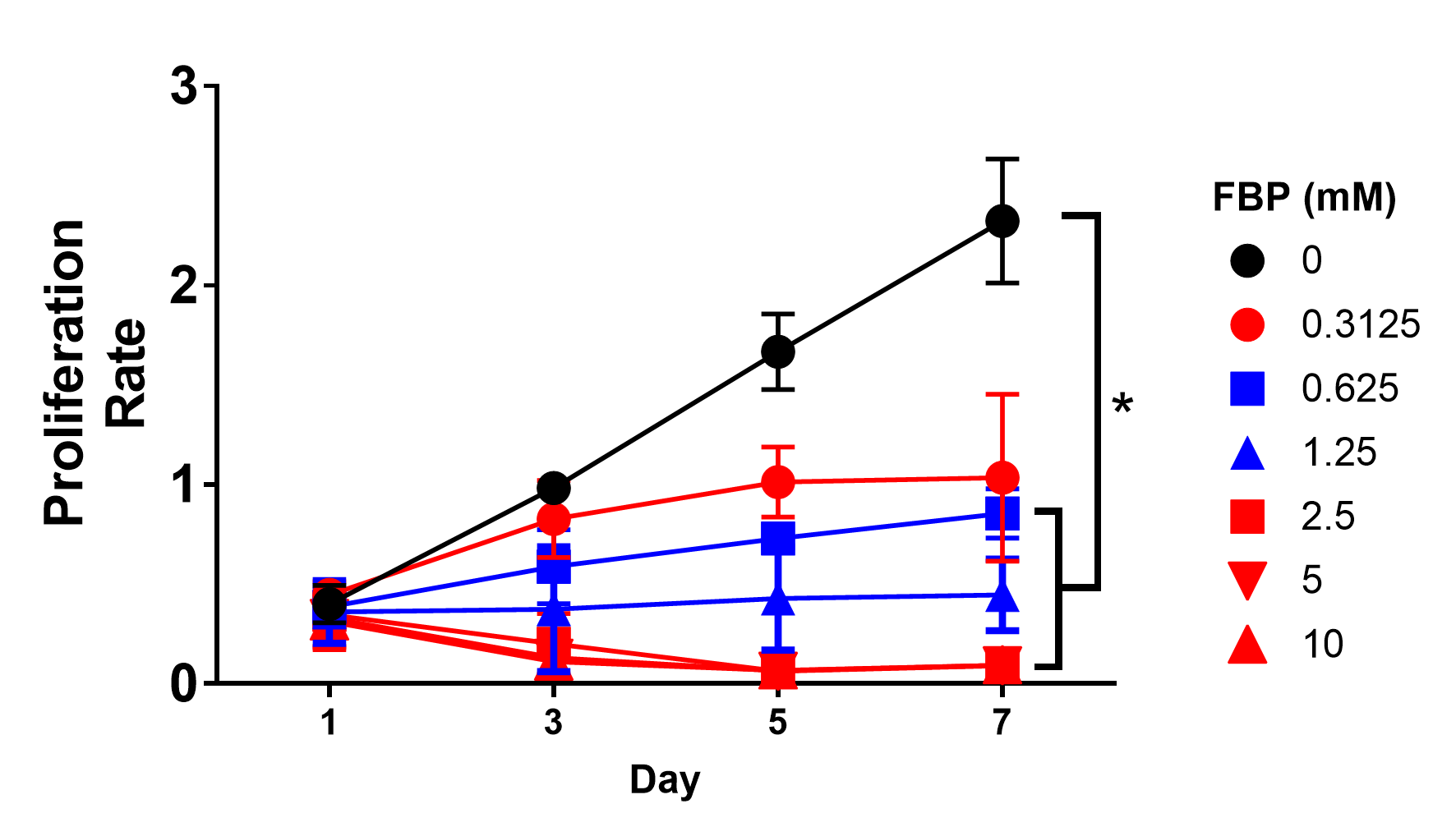

Supplement: S1 Fig — Cells were treated with 0 to 10 mM of FBP and proliferation rate of fibroblasts from healthy (HLF) lungs was assessed through 1 to 7 days after treatment. Y axis shows the proliferation rate as determined by MTT assay. Two-way ANOVA followed by Tukey’s multiple comparison test was used. Values are expressed as mean ± SD; n = 4–6/group; * p<0.05 determined on Day 3. (TIF) [file pone.0222202.s001.tif]
