# Supplementary material for: Fructose-1,6-bisphosphate prevents pulmonary fibrosis by regulating extracellular matrix deposition and inducing phenotype reversal of lung myofibroblasts
Source: PLoS One. 2019 Sep 11;14(9):e0222202. doi: 10.1371/journal.pone.0222202 (PMC6738633; doi:10.1371/journal.pone.0222202)
Supplement: S1 Table — (DOCX) [file pone.0222202.s002.docx]

**S1 Table. Primer sequences used for qRT-PCR analysis**

| **Gene** | **Primer Sequence** | |
| --- | --- | --- |
| *Cdh1* | Forward: | GAG GTC TAC ACC TTC CCG GT |
|  | Reverse: | AAA AGA AGG CTG TCC TTG GC |
| *Col1a1* | Forward: | GCT CCT CTT AGG GGC CAC T |
|  | Reverse: | CCA CGT CTC ACC ATT GGG G |
| *Col2a1* | Forward: | GGG AAT GTC CTC TGC GAT GAC |
|  | Reverse: | GAA GGG GAT CTC GGG GTT G |
| *Col4a1* | Forward: | GTC TGG CTT CTG CTG CTC TT |
|  | Reverse: | CAC ATT TTC CAC AGC CAG AG |
| *Col4a4* | Forward: | ATG AGG TGC TTT TTC AGA TGG AC |
|  | Reverse: | GGG GCC GCC ATA CTT CTT G |
| *Col4a6* | Forward: | ATC GGA TAC TCC TTC CTC ATG C |
|  | Reverse: | CCA GGG GAG ACT AGG GAC TG |
| *Col5a1* | Forward: | CTT CGC CGC TAC TCC TGT TC |
|  | Reverse: | CCC TGA GGG CAA ATT GTG AAA A |
| *Col5a2* | Forward: | TTG GAA ACC TTC TCC ATG TCA GA |
|  | Reverse: | TCC CCS GTG GGT GTT ATA GGA |
| *Col6a1* | Forward: | CTG CTG CTA CAA GCC TGC T |
|  | Reverse: | CCC CAT AAG GTT TCA GCC TCA |
| *Col6a2* | Forward: | AAG GCC CCA TTG GAT TCC C |
|  | Reverse: | CTC CCT TCC GAC CAT CCG AT |
| *Lox* | Forward: | TCT TCT GCT GCG TGA CAA CC |
|  | Reverse: | GAG AAA CCA GCT TGG AAC CAG |
| *Acta2* | Forward: | GCC GAG ATC TCA CCG ACT AC |
|  | Reverse: | ATG TCA CGG ACA ATC TCA CG |
| *Fn1* | Forward: | TTT GGC AGT GGT CAT TTC AG |
|  | Reverse: | CCC ACT TCT CTC CGA TCT TG |
| *Mmp2* | Forward: | CAA GTT CCC CGG CGA TGT C |
|  | Reverse: | TTC TGG TCS AGG TCA CCT GTC |
| *Cxcl12* | Forward: | TGC ATC AGT GAC GGT AAA CCA |
|  | Reverse: | TTC TTC AGC CGT GCA ACA ATC |
| *Sod1* | Forward: | AAG CGG TGA ACC AGT TGT G |
|  | Reverse: | CAT ACT GAT GGA CGT GGA ACC |
| *GAPDH* | Forward: | TGC GAC TTC AAC AGC AAC TC |
|  | Reverse: | CCT GTT GCT GTA GCC GTA TTC |
